# Cytokine and Antibody Based Diagnostic Algorithms for Sputum Culture-Positive Pulmonary Tuberculosis

**DOI:** 10.1371/journal.pone.0144705

**Published:** 2015-12-16

**Authors:** Tao Chen, Jinfei Lin, Wei Wang, Joy Fleming, Liang Chen, Yunxia Wang, Haicheng Li, Huixin Guo, Jie Zhou, Xunxun Chen, Yuhui Chen, Qinghua Liao, Yang Shu, Yaoju Tan, Meiling Yu, Guozhou Li, Lin Zhou, Qiu Zhong, Lijun Bi, Lina Guo, Meigui Zhao

**Affiliations:** 1 Center for Tuberculosis Control of Guangdong Province, Guangzhou, 510630, China; 2 Institute of Biophysics, Chinese Academy of Science, Beijing, 100101, China; 3 Foshan Fourth People’s Hospital, Foshan, 528000, China; 4 Bao’an Chronic Diseases Prevention and Cure Hospital, Shenzhen, 518102, China; 5 Guangzhou Chest Hospital, Guangzhou, 510095, China; 6 Chinese Medicine Hospital of Huangpu District, Guang zhou, 510700, China; 7 GuangDong Provincial Hospital of Chinese Medicine, GuangDong, 510120, China; 8 Chronic Disease Control and Prevention Station of Dongguan, Guangdong, 523008, China; Kings College London, UNITED KINGDOM

## Abstract

**Background:**

Tuberculosis (TB) is one of the most serious infectious diseases globally and has high mortality rates. A variety of diagnostic tests are available, yet none are wholly reliable. Serum cytokines, although significantly and frequently induced by different diseases and thus good biomarkers for disease diagnosis and prognosis, are not sufficiently disease-specific. TB-specific antibody detection, on the other hand, has been reported to be highly specific but not sufficiently sensitive. In this study, our aim was to improve the sensitivity and specificity of TB diagnosis by combining detection of TB-related cytokines and TB-specific antibodies in peripheral blood samples.

**Methods:**

TB-related serum cytokines were screened using a human cytokine array. TB-related cytokines and TB-specific antibodies were detected in parallel with microarray technology. The diagnostic performance of the new protocol for active TB was systematically compared with other traditional methods.

**Results:**

Here, we show that cytokines I-309, IL-8 and MIG are capable of distinguishing patients with active TB from healthy controls, patients with latent TB infection, and those with a range of other pulmonary diseases, and that these cytokines, and their presence alongside antibodies for TB-specific antigens Ag14-16kDa, Ag32kDa, Ag38kDa and Ag85B, are specific markers for active TB. The diagnostic protocol for active TB developed here, which combines the detection of three TB-related cytokines and TB-specific antibodies, is highly sensitive (91.03%), specific (90.77%) and accurate (90.87%).

**Conclusions:**

Our results show that combining detection of TB-related cytokines and TB-specific antibodies significantly enhances diagnostic accuracy for active TB, providing greater accuracy than conventional diagnostic methods such as interferon gamma release assays (IGRAs), TB antibody Colloidal Gold Assays and microbiological culture, and suggest that this diagnostic protocol has potential for clinical application.

## Introduction

Globally, tuberculosis (TB) is one of the most lethal infectious diseases, and requires long-term management. There are an estimated 9 million new cases per year, with 1.5 million deaths annually [1]. The relatively ineffectual control of the global epidemic of TB is partly due to the fact that 1/3 of the world’s population is thought to be infected with TB in a latent form, referred to as latent tuberculosis infection (LTBI)[2]. Approximately 5–10% of these LTBI cases may later develop into the active form of the disease. The most effective method of TB control is early diagnosis and intervention, but accurate detection of TB is hampered by the lack of simple, cost effective diagnostic tools.

The tuberculin skin test (TST) has been used for the detection of TB for many decades. It is defined as a delayed hypersensitivity response to purified protein derivative (PPD), a mixture of more than 200 *Mycobacterium tuberculosis* (Mtb) proteins [3]. This method is sensitive but not specific enough due to the inclusion of proteins from Bacillus Calmette-Guérin (BCG), the *Mycobacterium bovis* vaccination strain and many other environmental mycobacteria in PPD [4]. Currently, diagnosis of active tuberculosis is based on chest X-rays, and microscopy of at least three sputum samples. Culturing of *Mycobacterium tuberculosis* (Mtb) is required to provide a definitive diagnosis of TB [5]. Lowenstein-Jensen (LJ) culture is the most widely used medium for identifying characteristic features of colony morphology, growth rate, and pigment production. Though the technique is simple, its timeframe is extended, taking about 7–10 weeks, a factor which often leads to delays in diagnosis and treatment [6, 7]. Another diagnostic option is the BACTEC 960 liquid culture assay, an automated system that exploits fluorescence of an oxygen sensor to detect growth of mycobacteria. This assay reduces the time of detection to 9–14 days, but its application is limited by the high cost of instruments [8]. GeneXpert is a cartridge-based, automated diagnostic test that can identify *Mycobacterium tuberculosis* DNA and resistance to rifampicin (RIF) by a nucleic acid amplification test (NAAT).The concessional price for purchasing a GeneXpert system, however, is currently USD 17,000 for a four module instrument, and the cost of a test cartridge in countries eligible for concessional pricing is USD 9.98 (as of 6 August 2012) [9], significantly hampering it wide application in developing countries, especially for LTBI screening. In addition, current diagnostic methods do not detect the presence of *M*. *tuberculosis* in all cases, leading to the empirical treatment of many patients on the basis of clinical symptoms of active TB, and to the possibility that patients with other pulmonary diseases with similar symptoms are inaccurately diagnosed and treated for TB

An alternative immunodiagnostic approach, T-cell interferon gamma release assays (IGRAs), based on the reaction of cultured T-cells to early secretory antigen target-6 (ESAT-6) and culture filtrate protein-10 (CFP-10), has also been developed. These two antigens are strong targets of Th1 T-cells during Mtb infection [3]. However, IGRAs are unable to differentiate between active TB and LTBI, and are not suitable for treatment monitoring or determining recovery [10]. In addition, IGRAs are expensive and the protocol is complex.

Detection of Mtb-specific antibodies has been suggested as an important diagnostic aid [11]. Antibodies for many Mtb antigens have been suggested as biomarkers for active TB, including those for Ag38kDa, Ag16-kDa, ESAT-6, LAM, MPT63, Ag19-kDa, MPT32, MPT63, MPT64, MPT51, MTB48, Mtb39, Mtb81, MTC28, KatG and Ag85B [11–13]. The sensitivity and specificity of diagnosis with 38-kDa (Ag38kDa) and 16-kDa (Ag16) antigens is reported to be 52.5% and 93.3%, respectively [14]. Inclusion of MPT32 (MPT32, Ag32) with a polyprotein (Mtb11 + Mtb8 + Mtb48 + Mtb81) increases the sensitivity of diagnosis for active TB by 9% [15]. However, the poor overall sensitivity of this method limits its application in active TB diagnosis. To develop new diagnostic methods for active TB, attention has more recently focused on the detection of signals arising from the immune system reaction to Mtb activation, such as chemokine release by phagocytes, and antibodies against specific TB antigens. Some serum cytokines, including IP-10, IL-8, and MIG are reported to be stimulated after TB infection, and may be useful as diagnostic biomarkers [16–22]. IL-8, secreted by neutrophils, has been shown to be induced by Mtb [23] and enhances neutrophil killing of Mtb [24]. It is also involved in recruiting monocytes [25]. In a comparison of active TB patients and healthy controls, MIG secretion was significantly elevated in TB patients, in response to ESAT-6/CFP-10 stimulation [26–28].

In order to develop a new highly sensitive and specific method for fast serological diagnosis of active tuberculosis, we selected three cytokines (I-309, IL-8 and MIG) as biomarkers based on a quantitative cytokine array analysis, and developed an active TB diagnostic system which combines the detection of cytokines with that of antibodies for TB-specific antigens (Ag14-16kDa, Ag32kDa, Ag38kDa and Ag85B). The diagnostic protocol for active TB developed here, was highly sensitive, specific and accurate. Its diagnostic performance was significantly greater than conventional diagnostic methods such as interferon gamma release assays (IGRAs), TB antibody Colloidal Gold Assays and microbiological culture, strongly suggesting its potential for clinical application.

## Materials and Methods

### Ethics Statement

This study was reviewed and approved by the local ethics committee (Guangdong Center for Tuberculosis Control). Written informed consent was obtained from participants before they were enrolled in the study, and or from guardians on behalf of any juveniles who were enrolled.

### Sample Collection

In this study, 584 participants (Table 1) from Guangzhou, Shenzhen or Foshan, Guangdong Province, China, aged 13–65 years old, were enrolled. In total, 384 peripheral blood serum samples and 200 peripheral blood samples were collected from the 584 enrolled participants, which included 120 healthy controls, 45 individuals with latent TB infection (LTBI), 255 patients with active TB and 80 individuals with chronic pneumonia, lung cancer or other pulmonary diseases. The 120 healthy controls did not have any radiological or clinical signs of TB and had negative tuberculin skin test (TST) results (< 5 mm) and negative IGRA results against CFP-10, or ESAT-6, or both. The other 84 health controls did not have any radiological or clinical signs of TB and had negative tuberculin skin test (TST) results (< 5 mm), but without IGRA test. Individuals with allergic reactions, serious malnutrition, malignancy, or immunodeficiency, such as congenital immunodeficiency or HIV, and those receiving immunosuppressive therapy, were excluded. The 45 LTBI individuals were selected on the basis of a positive TST (> 10 mm) and a positive IGRA response against CFP-10, or ESAT-6, or both, in the absence of diagnostic criteria for active TB. These individuals mainly consisted of those with close patient contact or medical staff who worked at the Institute for TB Prevention and Treatment in Guangdong Province. The 255 participants with active TB disease were selected from an affiliated tuberculosis hospital, and were diagnosed on the basis of standard diagnostic criteria for pulmonary tuberculosis including classic symptoms (chronic cough with blood-tinged sputum, fever, night sweats, and weight loss), radiology (commonly chest X-rays), sputum microscopy and microbiological culture and strain identification. Samples were sex-matched. As the probability of a person coming into contact with Mtb increases over the course of a lifetime, it was considered more reliable to deem a younger individual testing TST-negative and IGRA-negative as TB-negative than an older individual with the same test results. The average age of the healthy control group in our study was thus lower than that of the LTBI group.

**Table 1:** Characteristics of the participants in the four groups.

Characteristic		Healthy controls	Latent TB infections	Active TB patients	Patients other pulmonary diseases
Total number		204	45	255	80
Female		111	20	100	33
Male		93	25	155	47
Ethnicity		Han Chinese	Han Chinese	Han Chinese	Han Chinese
Age (Mean Years ± SEM)		25.5 ± 9.06	38.00 ± 10.35	32.47 ± 12.68	40.47 ± 12.68
	Positive	0	45	200	0
TST	Negative	80	0	0	0
	Unknown	124	0	55	80
	Positive	0	45	73	0
IGRA	Negative	200	0	13	0
	Unknown	84	0	169	80
	Positive	0	0	255	0
LJ Culture	Negative	0	0	0	0
	Unknown	204	45	0	80

### TB serum biomarker screening with human cytokines arrays

Concentrations of 40 cytokines in the serum samples were measured with a commercial quantitative immuno-microarray (Quantibody Human Cytokine Array 1, RayBiotech, Inc., Norcross, GA, USA) according to the manufacturer’s instructions, and results were analyzed using the RayBiotech Q Analyzer program. Glass chips were washed after incubation in blocking buffer for 30 min, and each well was overlaid with 100 μL of diluted sample. After overnight incubation at 4°C and extensive washing, the chips were incubated for 1 h with detection antibody and then washed. AlexaFluor 555-conjugated streptavidin was then added and incubated for 0.5 h at room temperature. Signals (Cy3; 555 nm excitation, 655 nm emission) were scanned using a Genepix 4000B laser scanner (Axon Instruments, Foster City, CA, USA).

### ELISA and IGRA assays

ELISA assays were performed using human IL-8, MIG, and I-309 quantitative assay ELISA kits (R&D Corp.) according to the manufacturer’s instructions. IGRA assays were performed using *Mycobacterium tuberculosis* Specific Cell Mediated Immune Response Detection Kits (ELISA) (Hygeianey, Wuhan, China) according to the manufacturer’s instructions. Briefly, 1 ml fresh blood was stimulated with TB-specific antigens (ESAT-6 and CFP-10) or PBS (negative control) or lectins (positive control) for 20 h at 37°C in a water bath. Serum was collected after centrifugation at 4000g, and IFN-λ concentration was detected using a human IFN-λ ELISA kit. The results were read as Table 2:

**Table 2:** 

PBS (IU/ml)	Lectins-PBS (IU/ml)	TB-PBS (IU/ml)	Result
	any	≥0.35 and ≥ PBS/4	TB positive
	≥0.5	<0.35	TB negative
≤8	≥0.5	≥0.35 and < PBS/4	TB negative
	<0.5	<0.35	uncertainty
	<0.5	≥0.35 and < PBS/4	uncertainty
>8	any	any	uncertainty

### Mycobacterial detection and confirmation

Sputum microscopy and culture were used for mycobacterial detection, GenoType assay were used for positive culture confirmation. Suspicious sputa were digested using Nalc NaOH and concentrated by centrifugation, and then were used for Ziehl–Neelsen (ZN) staining followed by smear microscopy and inoculated into solid (Lowenstein–Jensen) and liquid (MGIT 960) culture media. Positive growths were confirmed for MTB by Para Nitro Benzoic acid test (PNB) and GenoType assay (Hain Diagnostika, Nehren, Germany).

### TB-specific IgG rapid tests

Human serum TB-specific antibodies were tested with TB antibody colloidal Gold Diagnostic kits. Kits were purchased from CTK BIOTECH (CA, USA) and assays were performed according to the manufacturer’s instructions. Serum specimens were assayed without any prior knowledge of the clinical status in every case.

### Human active TB diagnostic array development

**Fig 1:**
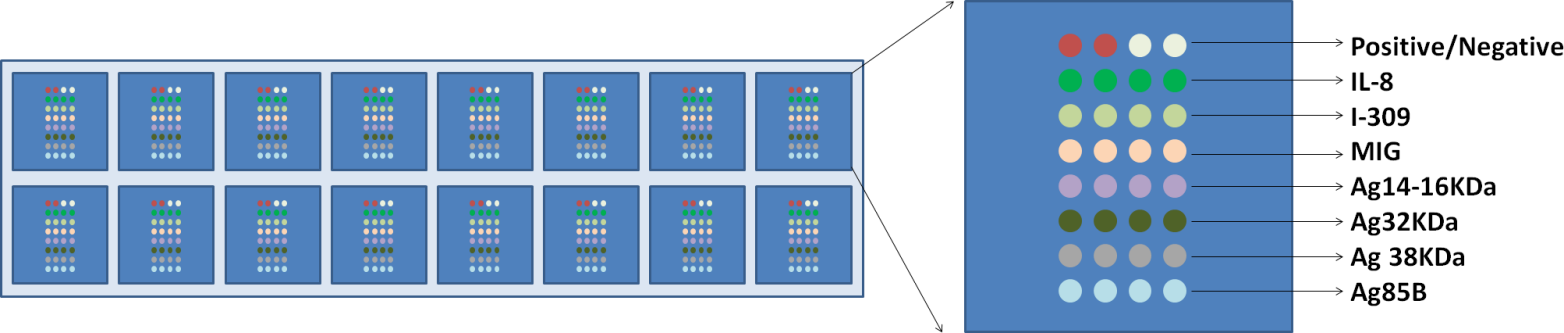
The arrangement of cytokines and antigens on TB-relate cytokine and TB-specific antigen array. Mouse anti-humane-IgG IgG was used as positive control and BSA were used as negative control.

Coating antibodies (mouse-anti-human IL8 IgG, mouse-anti-human MIG IgG, mouse-anti-human I-309 IgG; purchased from R&D, Minneapolis, MN, USA) and TB-specific antigens (Ag14-16kDa, Ag32kDa, Ag38kDa and Ag85B) were printed on a glass slide (ULTRAGAPS COATED SLIDES, Corning, US) with a Biodot 3210 printer (Biodot, Irvine, CA, USA). BSA was used as the negative control and mouse-anti-human IgG as the positive control. The array is shown in detail in Fig 1.

I-309, MIG and IL-8 standard antigens were purchased from R&D Systems (Minneapolis, MN, USA). To prepare antigen standards, 0.7 ng of I-309, 200 ng of MIG and 150 ng of IL-8 were added to 300 μl 5% BSA. The antigen standard was diluted 2-fold with 5% BSA. This dilution was repeated 5 times to create different concentrations of the antigen standard.

Arrays were blocked with 5% BSA for 2 h before incubating with either 100 μl of serum or differing concentrations of the antigen standard for 2 h at 37°C, then incubated with 100 μl of detection antibodies (biotin-anti-human IL-8, biotin-anti-human MIG, biotin-anti human I-309 and biotin- anti-human IgG at a final concentration of 20 ng/ml, purchased from R&D, Minneapolis, MN, USA) for 1 h at 37°C. Finally, arrays were incubated with 100 μl streptavidin-CY3 (1 ng/ml in PBS) for 30 min at 37°C. Between each of these steps, the slides were washed 5 times with PBST containing 0.5% Tween-20 and PBS. Slides were scanned with a Genepix 4000B laser scanner (Axon Instruments, Foster City, CA, USA).

A four-parameter logistic (4-PL) curve-fit was generated using SigmaPlot software. The lower limit of detection (LOD) for each marker in the assays was determined based on the average raw data of two sets of standard curves and from the average of two negative controls and their standard deviation (i.e., average + 2 × standard deviation). Signal strengths below the LOD for each biomarker in each assay were considered undetectable. For detection of TB-specific antibodies, if the signal was below the average of the negative control plus 3 times the SD, a negative result was recorded; otherwise, the result was recorded as positive. For the serum cytokines detection, if the concentration of the cytokine was above the cut-off value in Table 3, it was read as positive, or read as negative.

**Table 3:** Cut-off value for each cytokine.

Cytokines	Cut-off Value	AUC	Youden index J
I-309	0.04	0.895	0.7000
MIG	1.72	0.872	0.7000
IL-8	0.02	0.810	0.5833

### Statistical Analysis

Statistical analyses were conducted using ArrayTools statistical software. Significance analysis of microarrays (SAM) was used for class comparison and selection of target cytokines. ROC curve analysis was performed with SPSS20.0.

## Results

### Identification of I-309, IL-8 and MIG as serum markers for active TB

**Fig 2:**
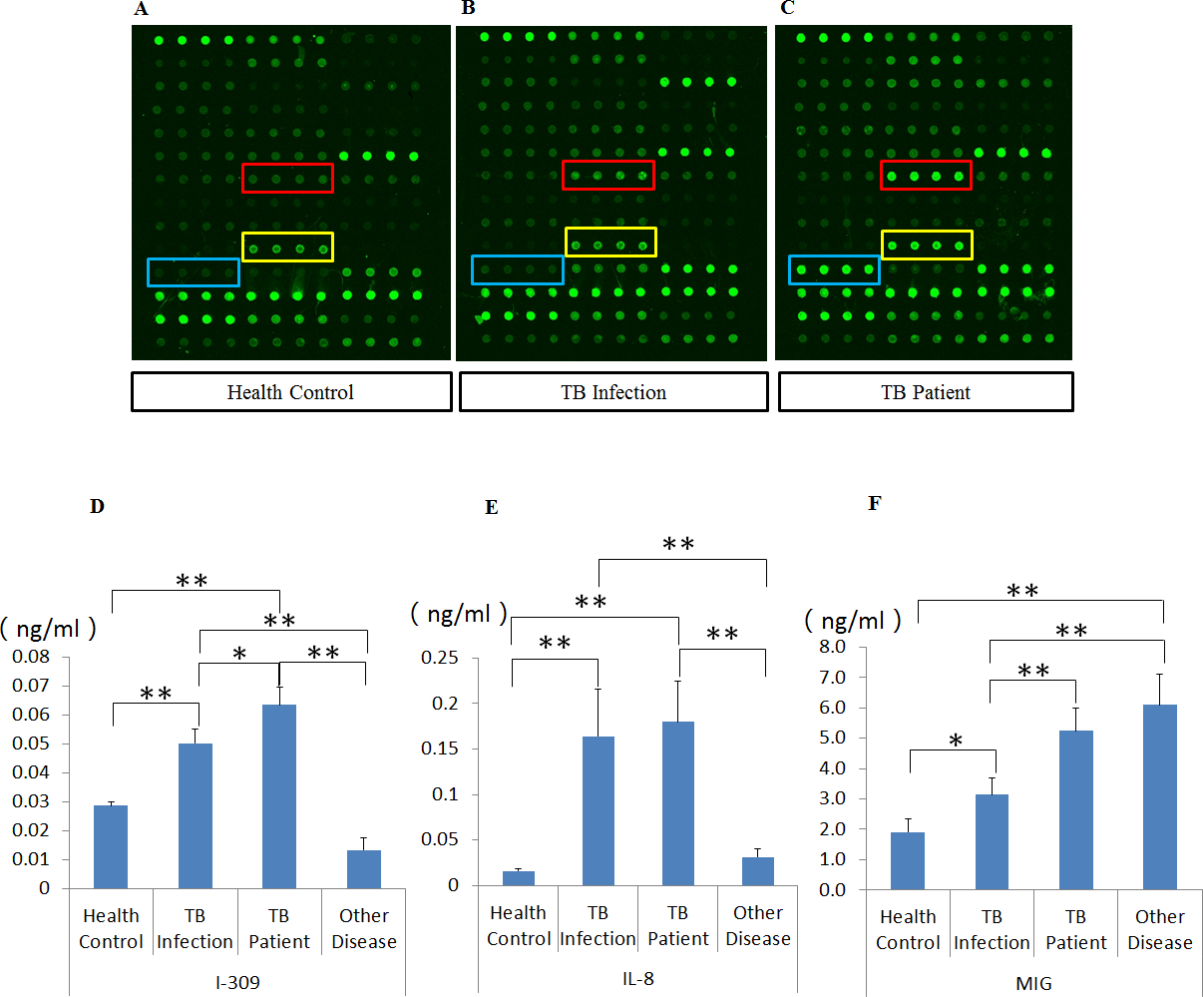
Up-regulation of I-309, IL-8, and MIG in the serum of active TB patients. (A-C) quantitative humane cytokine array analysis of peripheral serum samples from health controls (n = 20), individuals with LTBI (n = 20) and patients with active TB (n = 20). Blue, I-309; red, IL-8; yellow, MIG. (D-F) ELISA analysis for the confirmation of I-309, IL-8 and MIG expression in peripheral serum samples from health controls, patients with active TB, and individuals with LTBI or other pulmonary diseases (n = 45 in each group). Mean values ± standard error are shown, * P<0.05; ** P < 0.01 (Student’s *t*-test).

To screen for serum cytokine biomarkers for TB, we performed a quantitative cytokine array analysis of peripheral serum samples from healthy controls (N), and individuals with LTBI (I) or active TB (P, TB culture test positive). We found that of the 40 human cytokines tested, I-309, IL-8 and MIG, were all significantly upregulated in patients with active TB (from 2.63–5.16 fold, p<0.01) compared to the other two groups (Fig 2A–2C, and data not shown). ELISA analysis of peripheral serum samples from healthy controls, and individuals with LTBI, active TB or other pulmonary diseases confirmed these results (Fig 2D–2F). Both I-309 and IL-8 were more highly expressed in patients with active TB than in the other three groups (p<0.01), and MIG was upregulated in patients with active TB and subjects with other pulmonary diseases, compared to the healthy control and LTBI groups.

**Fig 3:**
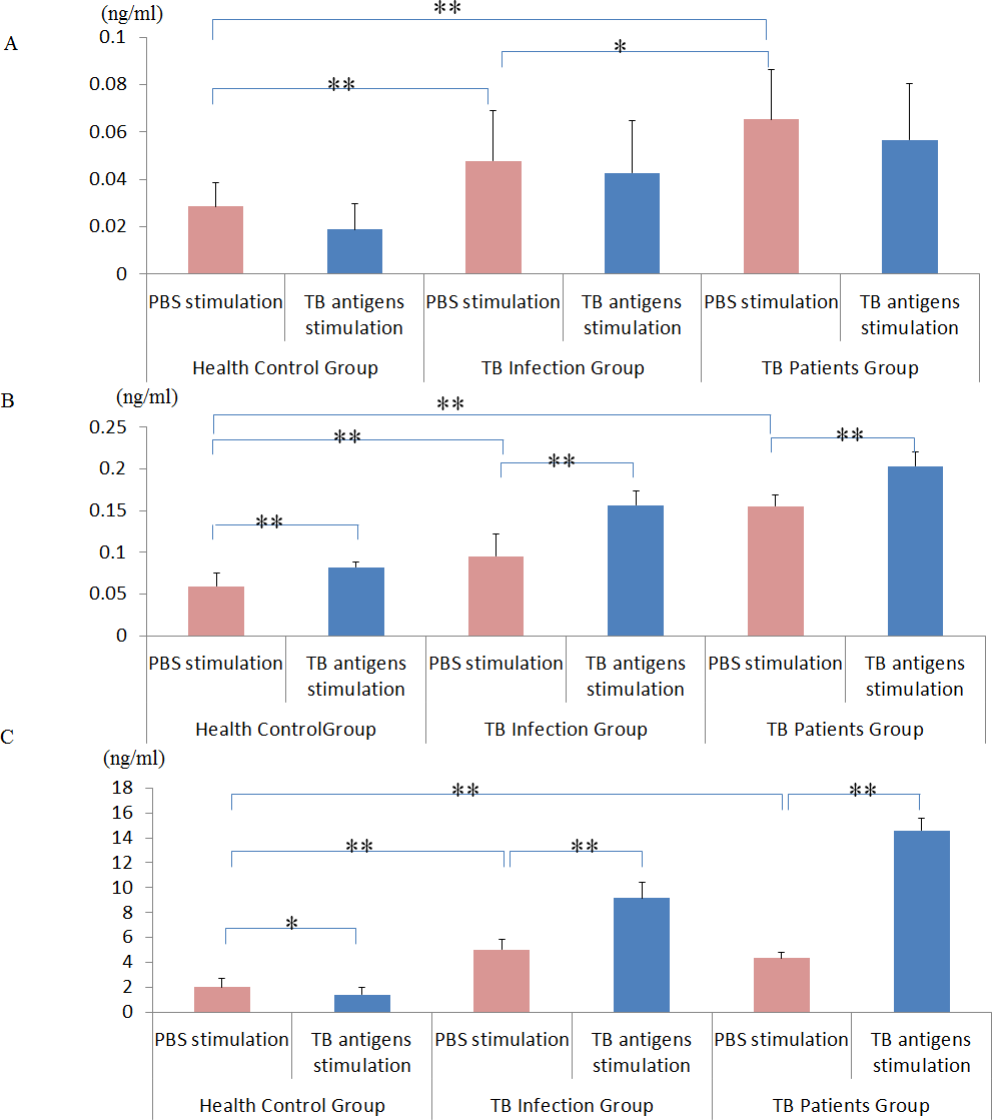
I-309, IL-8 and MIG over-expression is stimulated by Mtb antigens (ESAT-6 and CFP-10) in active TB. Expression of I-309 (A), IL-8 (B), and MIG (C) induced by PBS or specific TB antigens in whole blood displayed a gradual increase from health controls (n = 80) to individuals with LTBI (n = 45), to those with active TB (n = 80). Mean values ± standard error are shown, *P < 0.05; **P < 0.01 (Student’s *t*-test).

In order to show that I-309, IL-8 and MIG are released by lymphocytes in response to TB infection, we incubated TB-specific antigens (ESAT-6 and CFP-10) with whole blood samples from healthy controls, or individuals with LTBI or active TB (TB culture test positive). Expression of each of these three cytokines in whole blood samples was significantly higher in individuals with active TB than in those with LTBI and healthy controls (active TB > LTBI > healthy controls; Fig 3). The expression of I-309 was stable after TB antigen stimulation (Fig 3A) in all groups. IL-8 expression was more significantly upregulated when stimulated with TB antigens than when incubated with PBS in healthy controls and individuals with LTBI or active TB (Fig 3B). The expression of MIG was significantly higher in individuals with LTBI or active TB, but was lower in healthy controls (Fig 3C).

### Evaluation of I-309, IL-8 and MIG for diagnosis of active TB

**Fig 4:**
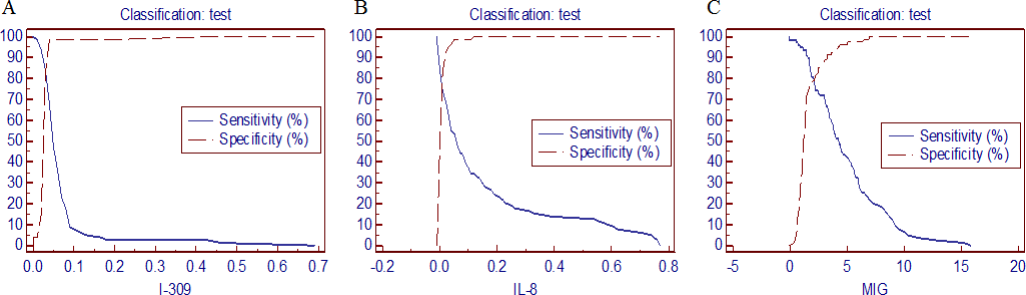
Determination of cut-off values for I-309, IL-8 and MIG in the diagnosis of active TB.

To evaluate the efficiency of I-309, IL-8 and MIG in diagnosing active TB, peripheral serum samples from healthy control subjects, and individuals with active TB (TB culture test positive) or other pulmonary diseases (n = 81 in each group) were tested by quantitative cytokine array analysis, and 95% CIs were calculated for the three groups (Table 4). A ROC analysis was applied to determine the cut-off value of I-309, IL-8 and MIG (Fig 4). At cut-off values of 0.04, 0.02 and 1.72 ng/ml, respectively, an optimal balance of specificity and sensitivity was achieved, giving Yonden Indexes of 0.7, 0.5833, and 0.7, respectively (Table 3). ROC curve analysis gave AUC values of 0.895, 0.810 and 0.872, respectively, indicating the potential of these three cytokines as serum biomarkers.

**Table 4:** The expression level of I-309, MIG and IL-8 in the peripheral serum of human from different group.

Group	Mean/SD	I309	MIG	IL-8
		(ng/ml)	(ng/ml)	(ng/ml)
	$\bar{\text{x}}$	0.0306	1.6676	0.0119
Normal	SD	0.0053	1.3903	0.0169
	95% CI	0.029–0.0319	1.3084–2.0267	0.0076–0.0163
	$\bar{\text{x}}$	0.079	5.1251	0.1716
Patients	SD	0.1002	3.4542	0.2434
	95% CI	0.0531–0.1049	4.2328–6.0174	0.1088–0.2345
	$\bar{\text{x}}$	0.046	5.2633	0.0171
Other pulmonary diseases	SD	0.3454	4.4453	0.0331
	95% CI	0.0645–0.1565	3.8416–6.6849	0.0065–0.0277

### Evaluation of TB-specific antigens for active TB diagnosis

In previous studies, the Ag14-16kDa [29], Ag32kDa [30], Ag38kDa [31, 32], and Ag85B [33]antigens were reported to be sensitive and specific for TB antibody detection in serum samples. Here we detected antibodies for these antigens in peripheral serum samples from healthy controls, individuals with active TB (TB culture test positive), or other pulmonary diseases (n = 81 in each group) using ELISpot. Sensitivity for each antigen was moderate (45.0%, 53.3%, 28.3% and 55.0%, respectively), while specificity was high (97.5%, 91.7%, 100.0% and 93.3%, respectively) (Table 5). Two methods were used to interpret results. In method A, a diagnosis of active TB was given if any one of the four TB-specific antigens was detected (Table 6). In method B, a diagnosis of active TB was given if three of the four antigens were detected (Table 7). The effectiveness of the two methods is compared in Table 8: method A was more efficient for diagnosis and had relatively high sensitivity (71.7%) and specificity (89.1%).

**Table 5:** Evaluation of TB-specific antigens using clinical samples.

TB-specific antigens	Test result	Active TB patients	Patients with other pulmonary diseases	Health controls	Sensitivity (%)	Specificity (%)
	Positive	27	2	1		
Ag14-16KD	Negative	32	58	59	45	97.5
	Total	60	60	60		
	Positive	32	5	5		
Ag 32KD	Negative	28	55	55	53.3	91.7
	Total	60	60	60		
	Positive	17	0	0		
Ag 38KD	Negative	43	60	60	28.3	100
	Total	60	60	60		
	Positive	33	5	3		
Ag 85B	Negative	27	55	57	55	93.3
	Total	60	60	60		

**Table 6:** Diagnostic results for TB specific antigens Method A.

**Test result**	**Active TB patients**	**Patients with other pulmonary diseases**	**Health controls**	**Total**
**Positive**	43	8	5	56
**Negative**	17	52	55	124
**Total**	60	60	60	180

**Table 7:** Diagnostic results for Method B.

**Test result**	**Active TB patients**	**Patients with other pulmonary diseases**	**Health controls**	**Total**
**Positive**	10	0	0	10
**Negative**	50	60	60	170
**Total**	60	60	60	180

**Table 8:** Comparison of the effectiveness of Methods A and B.

**Method**	**Sensitivity (%)**	**Specificity (%)**	**Accuracy (%)**	**False positive (%)**	**Sensitivity (%)**	**PPV (%)**	**NPV (%)**
**A**	71.7	89.1	83.3	10.8	28.3	76.8	86.3
**B**	16.7	100	81.5	0	83.3	100	70.6

### Diagnosis of active TB using a combination of TB-related cytokine and TB-specific antibody detection

Based on the above findings, we developed a combined TB-related cytokine and antigen array for detecting active TB (TB culture test positive). The array enables quantitative detection of I-309, IL-8 and MIG and TB-specific IgGs, using a single small tube. Diagnostic criteria for active TB for use with the array are: positive expression of I-309, IL-8 and MIG above the cut-off value, or detection of antibodies for any one of the four antigens: Ag14-16kDa, Ag32kDa, Ag38kDa, and Ag85B. To test its efficiency for diagnosing active TB, we used the array to test clinical peripheral serum samples from healthy controls, individuals with active TB or other pulmonary diseases (Table 9). Combining cytokine and TB-specific antigen detection gave high sensitivity and specificity for distinguishing active TB patients from both healthy controls (91.03%, 92.59%, Table 10) and other pulmonary diseases (91.03%, 87.76%, Table 11). It proved very efficient for the diagnosis of active TB, with 91.03% sensitivity and 90.77% specificity (Table 12). Although detection of cytokines or antigens alone is more specific for active TB, combining the two factors significantly increased the sensitivity from 56.41% for cytokine detection and 71.79% for antigen detection to 91.03% (Table 12).

**Table 9:** Evaluation of the discriminatory power of our cytokine and antigen array for distinguishing active TB patients from health controls and individuals with other pulmonary diseases.

Marker combination	Test result	Health controls	Active TB patients	Patients with other pulmonary diseases
	Positive	0	44	1
I-309+MIG+IL-8	Negative	81	34	48
	Total	81	78	49
	Positive	6	56	5
38KDa+32KDa+14-16KDa+Ag85B	Negative	75	22	44
	Total	81	78	49
	Positive	6	71	6
I-309+MIG+IL-8+ 38KDa +32KDa+14-16KDa+Ag85B	Negative	75	7	43
	Total	81	78	49

**Table 10:** The efficacy of our cytokine and antigen array for distinguishing active TB patients from heathy controls.

Marker combination	Sensitivity (%)	Specificity (%)	PPV (%)	NPV (%)	Accuracy (%)	Youden index (%)
I-309+MIG+IL-8	56.41	100	100	70.43	78.62	56.41
38KDa+32KDa+14-16KDa+85B	71.79	92.59	90.32	77.32	82.39	64.39
I-309+MIG+IL-8+ 38KDa +32KDa+14-16KDa+Ag85B	91.03	92.59	92.21	91.46	91.82	83.62

**Table 11:** The performance of TB relate cytokine and TB-specific antigen array for distinguishing active TB patients from individuals with other pulmonary diseases.

Marker combinations	Sensitivity (%)	Specificity (%)	PPV (%)	NPV (%)	Accuracy (%)	Youden index (%)
I-309+MIG+IL-8	56.41	97.96	97.78	58.54	72.44	54.37
38KDa+32KDa+14-16KDa+85B	71.79	89.80	91.80	66.67	78.74	61.59
I-309+MIG+IL-8+ 38KDa +32KDa+14-16KDa+Ag85B	91.03	87.76	92.21	86.00	89.76	78.78

**Table 12:** The performance of TB relate cytokine and antigen array for distinguishing active TB patients from both health controls and individuals with other pulmonary diseases.

Marker combinations	Sensitivity (%)	Specificity (%)	PPV (%)	NPV (%)	Accuracy (%)	Youden index (%)
I-309+MIG+IL-8	56.41	99.23	97.78	79.14	83.17	55.64
38KDa+32KDa+14-16KDa+Ag85B	71.79	91.54	83.58	84.4	84.13	63.33
I-309+MIG+IL-8+38KDa +32KDa+14-16KDa+Ag85B	91.03	90.77	85.54	94.4	90.87	81.79

### Comparison of our new TB diagnostic method with other current TB diagnostic methods

In order to evaluate the advantages and applicability of our TB array for active TB diagnosis, we compared its diagnostic performance with conventional diagnostic methods such as IGRAs, TB antibody Colloidal Gold Assays and microbiological culture using a larger clinical sample population (n = 405; 201 TB patients and 204 healthy controls). The sensitivity, specificity and accuracy of our method for detecting active TB was 91.03%, 92.59 and 91.82%, respectively. This compares very favorably with other widely used methods such as IGRAs (84.88%, 76.47% and 82.50%), TB Antibody Colloidal Gold Assays (69.65%, 60.78% and 65.19%) and microbiological culture (35.32%, 100% and 67.90%) (Table 13).

**Table 13:** Comparison of the performance of different TB diagnostic methods.

Diagnostic Method		Active TB patients	Health controls	Sensitivity (%)	Specificity (%)	PPV (%)	NPV (%)	Accuracy (%)
Cytokine / Antigen array	Positive	71	6	91.03	92.59	92.21	91.46	91.82
	Negative	7	75					
IGR assay	Positive	73	8	84.88	76.47	90.12	66.67	82.5
	Negative	13	26					
TB-specific antibody test (Colloidal Gold)	Positive	140	80	69.65	60.78	63.63	67.03	65.19
	Negative	61	124					
LJ culture	Positive	71	0	35.32	100	100	61.08	67.9
	Negative	130	204					

## Discussion

Serum cytokines, although significantly and frequently induced by different diseases and thus good biomarkers for disease diagnosis and prognosis, are not sufficiently disease-specific. TB-specific antibody detection, on the other hand, has been reported to be highly specific but not sufficiently sensitive. In this study, our aim was to improve the sensitivity and specificity of TB diagnosis by combining detection of cytokines (I-309, IL-8 and MIG) and TB-related antigens (Ag14-16kDa, Ag32kDa, Ag38kDa, and Ag85B) in peripheral blood samples.

I-309, IL-8 and MIG were identified as biomarkers for active TB in a previously reported quantitative cytokine array analysis of peripheral serum samples from healthy controls, LTBI patients and active TB patients. Interestingly, although levels of these cytokines vary in response to a range of diseases, a simultaneous increase in all three cytokines appears to be a signature of active TB disease. ELISA assays confirmed that I-309, IL-8 and MIG expression are significantly up-regulated compared with healthy controls and patients with other pulmonary diseases (Fig 2D, 2E and 2F). I-309 is an inflammatory mediator that specifically stimulates human monocytes and is secreted by activated T lymphocytes [34]. IL-8 is a member of the CXC family and is mostly produced by macrophages and neutrophils to enhance neutrophil killing and to recruit monocytes [25]. MIG is a chemoattractant that activates T lymphocytes [35]. The serum concentrations of these three cytokines increased progressively here from bacteria-free individuals to LTBI patients and active TB patients (Fig 2D, 2E and 2F), suggesting that these three cytokines may play a role in activating the host defense response in TB. In another study, we found that the concentration of MIG decreased gradually during chemotherapy, and was significantly lower in patients who had recovered from TB (unpublished data). Thus, MIG may have potential as an indicator for prognosis or drug efficacy.

Antibodies for TB-specific antigens have previously been suggested as biomarkers; however, to date, the sensitivity of known single antigens is not high enough to replace sputum smear microscopy [36]. In this study, we have used four antigens together to diagnose active TB. We examined levels of Ag14-16kDa, Ag32kDa, Ag38kDa, and Ag85B in peripheral serum samples from healthy controls, and individuals with active TB or other pulmonary diseases. Our data show that detection of any one of these four antigens can indicate the presence of TB (Table 5). This approach is more sensitive for diagnosing active TB than testing for the co-expression of three of the antigens, possibly because recognition of antigens is heterogeneous [37]; due to differences in bacillary load, the specific stage of disease or the immunogenetic background of the patient, antibodies presenting in peripheral blood can present a wide range of patterns. Of the Mtb-specific antigens that have been suggested as biomarkers for active TB, the 16-kDa antigen is a member of the low molecular weight heat-shock protein family [38, 39]. It is reported to be a prominent Mtb antigen whose epitopes, based on B-cell recognition, are restricted to the *M*. *tuberculosis* complex [29]. Ag32kDa is homologous to a fibronectin-binding protein of *Mycobacterium leprae* (43 L) believed to be involved in the invasion of epithelial and Schwann cells [40], and it has strong reactivity with serum antibodies in TB patients [41]. The 38-kDa protein is the most widely studied antigen of TB because it offers >95% species-specificity. Recognition frequency reported for the 38-kDa antigen varies greatly (16−94%) with smear status and disease manifestation [14, 42, 43]. Ag85B is a component of the Antigen85 complex, the early-secreted antigens of the major secretory proteins of Mtb [33, 44]. It is likely that this antigen can be recognized by the host system during the early stages of the disease [12]. It has been suggested that these antigens may share antigenic epitopes with the BCG vaccine and that BCG vaccination may interfere with the detection of antibody responses to these antigens [11]. However, the immune response due to vaccination with BCG early in life is unlikely to influence the antibody response observed here [45, 46].

In clinical practice, sputum is still the most important sample used in laboratory testing. However, only 44% of all new cases can be identified by the presence of acid-fast bacilli (AFB) in sputum smears [47]. While microbiological culture is the gold standard for diagnosis of active TB, culturing Mtb requires more than two weeks on average [6, 7, 48]. Chest X-rays are still the most widely used method for diagnosing and monitoring treatment responses in TB patients. However, chest X-rays are not specific for pulmonary TB, and thus cannot provide a conclusive diagnosis on their own [49]. Chest computed tomography (CT), especially high-resolution CT, is more sensitive than the chest X-ray [50], however, it is too expensive to be used widely in developing countries. The Mtb-specific nucleic acid amplification test (NAAT) for bronchopulmonary specimens is a newly developed molecular test for laboratory diagnosis of TB. NAAT is simple and fast, and results are available within a single day. However, the price is too high for wide application and diagnostic accuracy in smear-negative patients is controversial [51, 52].

By combining the two assays, we have developed an efficacious diagnostic system for active TB. Positive expression of I-309, IL-8 and MIG above the cut-off value, or detection of antibodies for any one of the four antigens, Ag14-16kDa, Ag32kDa, Ag38kDa, and Ag85B, provide stronger diagnostic criteria for active TB than other widely used methods. This approach is both highly sensitive (91.03%), specific (92.59%) and accurate (91.82%). Our diagnostic system for active TB uses peripheral serum, the most common clinical sample, and the system is operationally simple and can be completed within less than four hours. All these features of our diagnostic system for active TB suggest its efficacy for clinical application. However, results obtained here need to be further verified with a larger sample population before this new diagnostic protocol can be developed into a clinical diagnostic test for active TB. Further validation is also necessary to determine whether the chips developed here can be used to distinguish between active and latent tuberculosis. In addition, the selection of the three cytokines evaluated here was based on results obtained using a commercial human cytokine array which detects 40 common human cytokines in serum. It is possible that there are additional, or perhaps even more suitable, serum cytokines that could also be used for auxiliary diagnostic tests for TB. These issues will be resolved in future studies on TB diagnostics in our lab.

## Conclusions

The combining detection of TB-relate cytokines and TB-specific antibodies could significantly enhance active TB diagnosis accuracy compared with conventional diagnostic methods such as interferon gamma release assays (IGRAs), TB antibody Colloidal Gold Assays and microbiological culture. Which strongly suggest its potential for clinical application, especially for sputum smear negative/ culture negative patient’s diagnosis, which could be probably reduce the frequency and impact of unnecessary empiric treatment.
